# Supplementary figures and images for: Diversity, composition, and networking of saliva microbiota distinguish the severity of COVID-19 episodes as revealed by an analysis of 16S rRNA variable V1-V3 region sequences
Source: mSystems. 2023 Jun 13;8(4):e01062-22. doi: 10.1128/msystems.01062-22 (PMC10470033; doi:10.1128/msystems.01062-22)

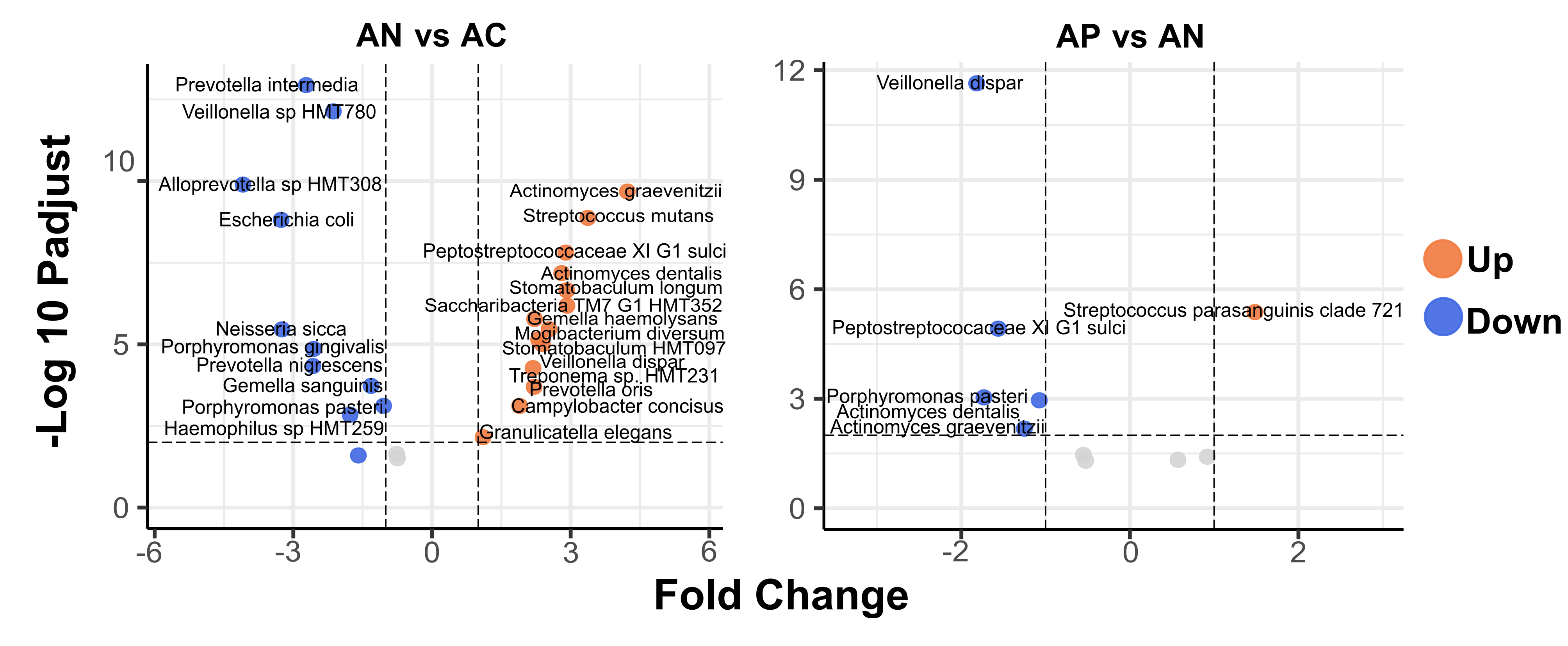

Supplement: Fig. S1 — Volcano analysis of microbiota in patients with mild disease. [file msystems.01062-22-s0001.tif]

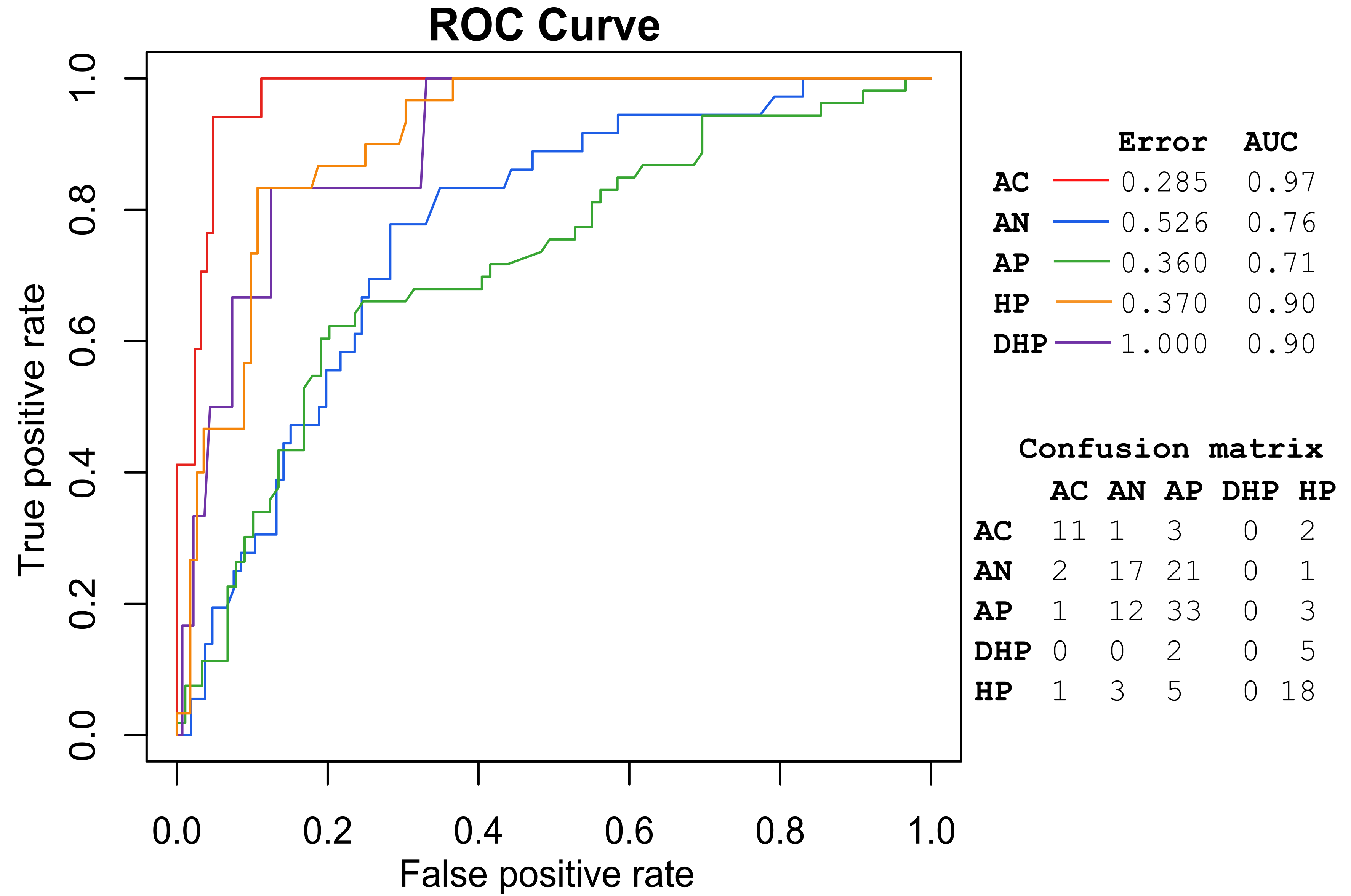

Supplement: Fig. S2 — ROC curve showing significance in microbial composition between group. [file msystems.01062-22-s0002.tif]

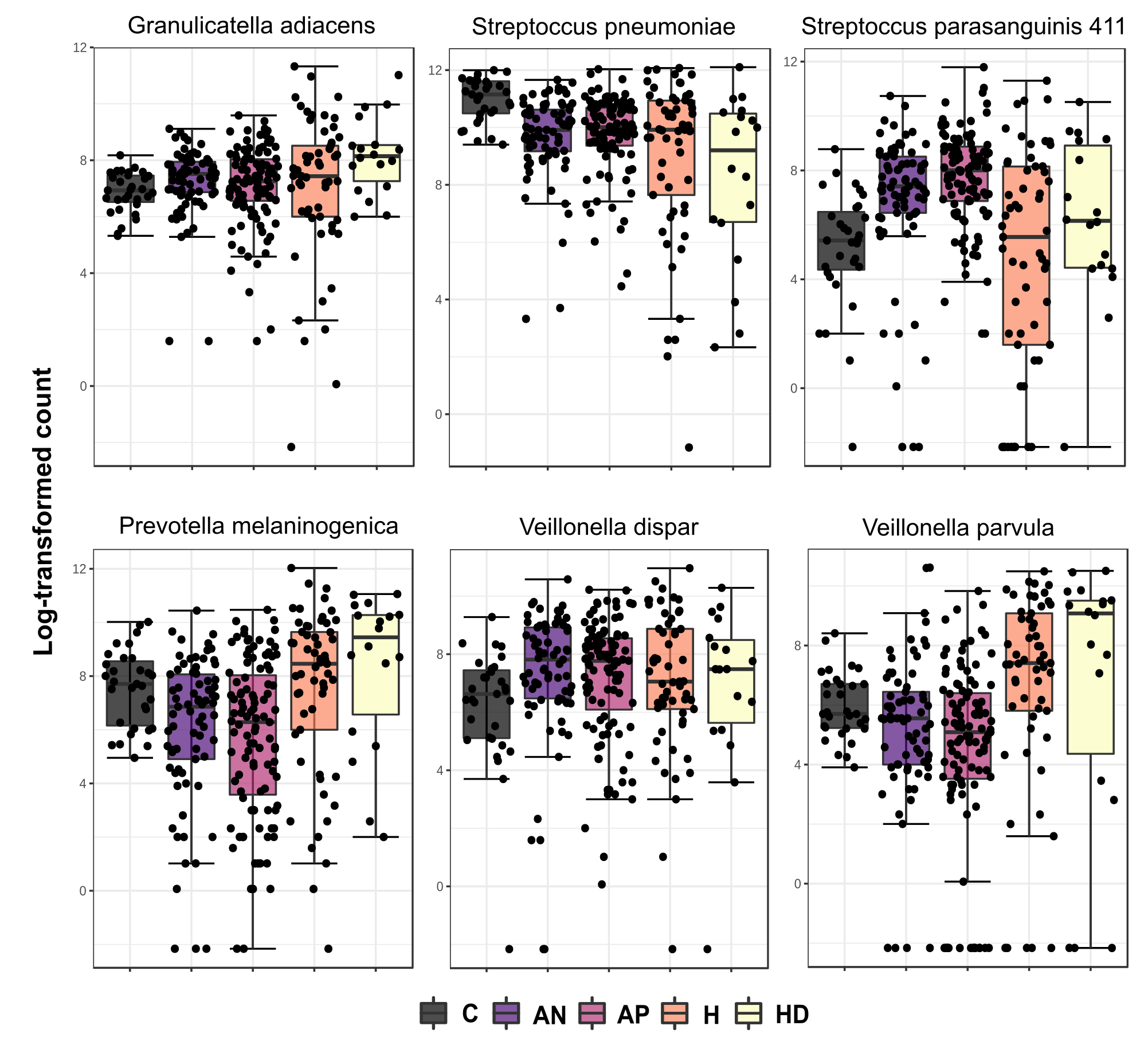

Supplement: Fig. S3 — Relative abundance of species significantly different between groups. [file msystems.01062-22-s0003.tif]

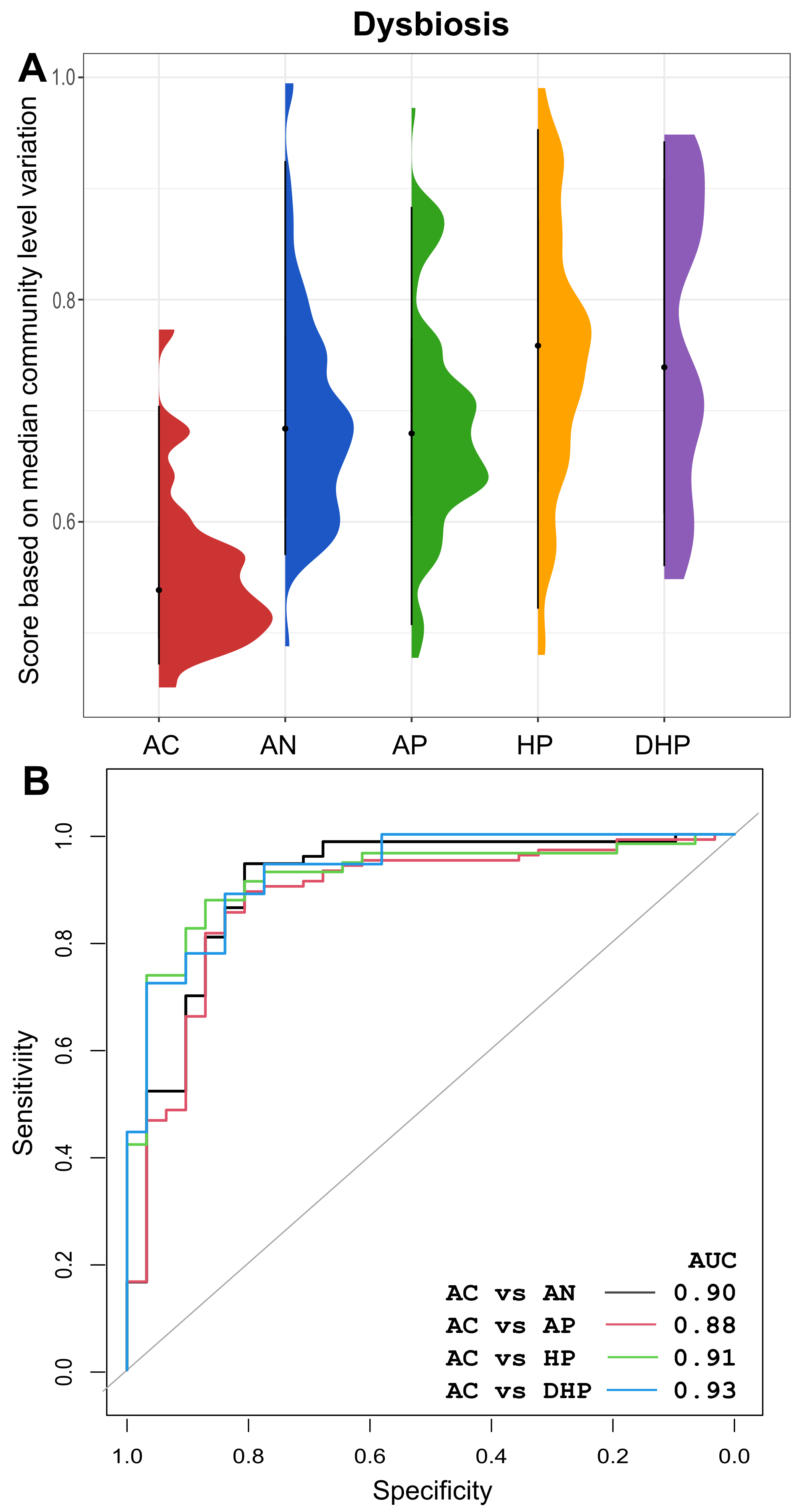

Supplement: Fig. S4 — Score of dysbiosis in symptomatic patients SARS-CoV-2 infected. [file msystems.01062-22-s0004.tif]

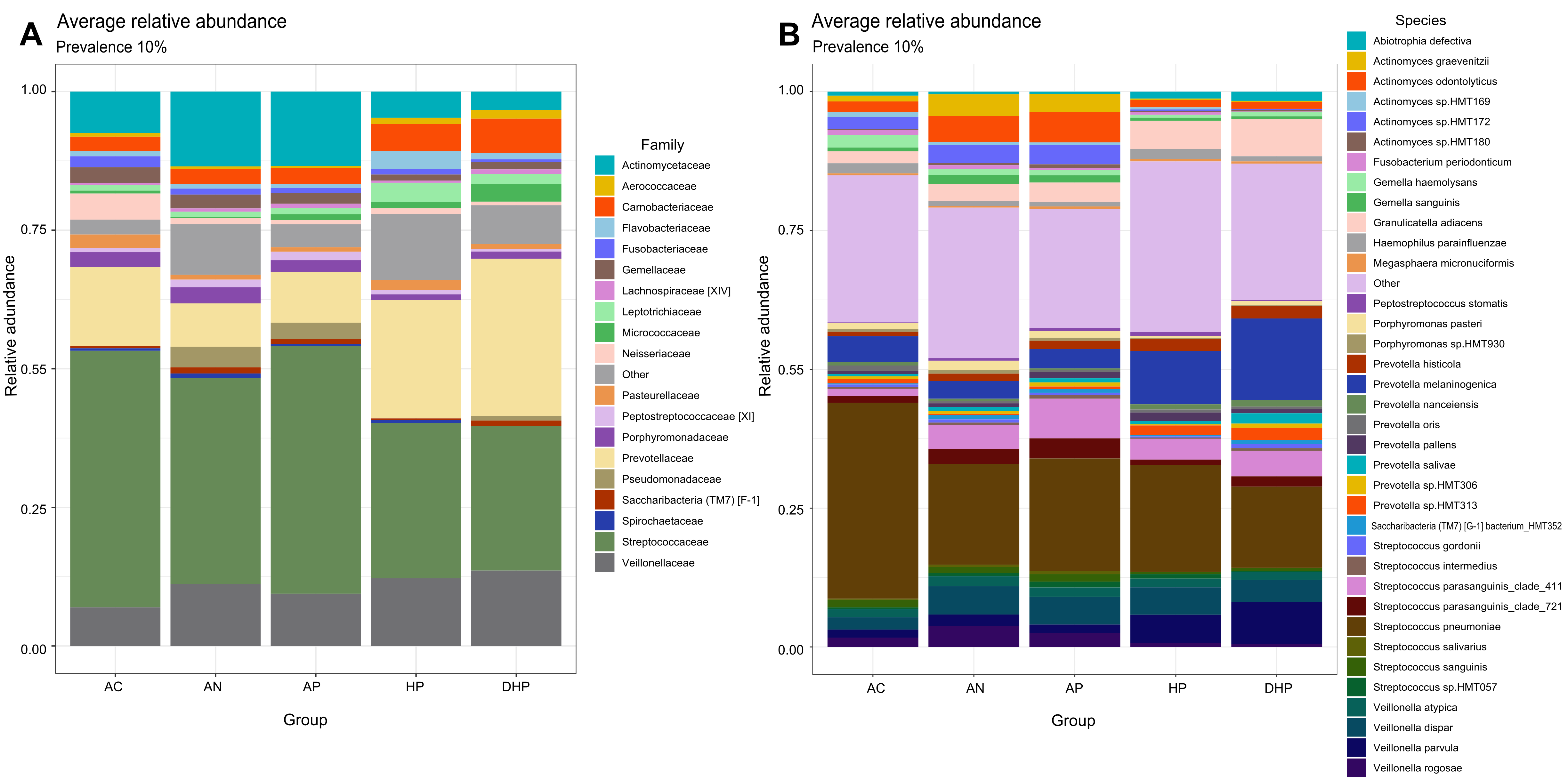

Supplement: Fig. S5 — Stack bar of taxa in each clinical group at the level of family and species. [file msystems.01062-22-s0005.tif]
