# Supplementary material for: Diversity, composition, and networking of saliva microbiota distinguish the severity of COVID-19 episodes as revealed by an analysis of 16S rRNA variable V1-V3 region sequences
Source: mSystems. 2023 Jun 13;8(4):e01062-22. doi: 10.1128/msystems.01062-22 (PMC10470033; doi:10.1128/msystems.01062-22)
Supplement: Table S1 — List of amplification primers. [file msystems.01062-22-s0006.docx]

| Name | Sequence 5´- 3´ |
| --- | --- |
| FW.A501 | AATGATACGGCGACCACCGAGATCTACACATCGTACGTATGGTAATTCAATTACCGCGGCTGCTGG |
| FW.A502 | AATGATACGGCGACCACCGAGATCTACACACTATCTGTATGGTAATTCAATTACCGCGGCTGCTGG |
| FW.A503 | AATGATACGGCGACCACCGAGATCTACACTAGCGAGTTATGGTAATTCAATTACCGCGGCTGCTGG |
| FW.A504 | AATGATACGGCGACCACCGAGATCTACACCTGCGTGTTATGGTAATTCAATTACCGCGGCTGCTGG |
| FW.A505 | AATGATACGGCGACCACCGAGATCTACACTCATCGAGTATGGTAATTCAATTACCGCGGCTGCTGG |
| FW.A506 | AATGATACGGCGACCACCGAGATCTACACCGTGAGTGTATGGTAATTCAATTACCGCGGCTGCTGG |
| FW.A507 | AATGATACGGCGACCACCGAGATCTACACGGATATCTTATGGTAATTCAATTACCGCGGCTGCTGG |
| FW.A508 | AATGATACGGCGACCACCGAGATCTACACGACACCGTTATGGTAATTCAATTACCGCGGCTGCTGG |
| FW.B501 | AATGATACGGCGACCACCGAGATCTACACCTACTATATATGGTAATTCAATTACCGCGGCTGCTGG |
| FW.B502 | AATGATACGGCGACCACCGAGATCTACACCGTTACTATATGGTAATTCAATTACCGCGGCTGCTGG |
| RE.A701 | CAAGCAGAAGACGGCATACGAGATAACTCTCGAGTCAGTCAGCCGAGTTTGATCMTGGCTCAG |
| RE.A702 | CAAGCAGAAGACGGCATACGAGATACTATGTCAGTCAGTCAGCCGAGTTTGATCMTGGCTCAG |
| RE.A703 | CAAGCAGAAGACGGCATACGAGATAGTAGCGTAGTCAGTCAGCCGAGTTTGATCMTGGCTCAG |
| RE.A704 | CAAGCAGAAGACGGCATACGAGATCAGTGAGTAGTCAGTCAGCCGAGTTTGATCMTGGCTCAG |
| RE.A705 | CAAGCAGAAGACGGCATACGAGATCGTACTCAAGTCAGTCAGCCGAGTTTGATCMTGGCTCAG |
| RE.A706 | CAAGCAGAAGACGGCATACGAGATCTACGCAGAGTCAGTCAGCCGAGTTTGATCMTGGCTCAG |
| RE.A707 | CAAGCAGAAGACGGCATACGAGATGGAGACTAAGTCAGTCAGCCGAGTTTGATCMTGGCTCAG |
| RE.A708 | CAAGCAGAAGACGGCATACGAGATGTCGCTCGAGTCAGTCAGCCGAGTTTGATCMTGGCTCAG |
| RE.A709 | CAAGCAGAAGACGGCATACGAGATGTCGTAGTAGTCAGTCAGCCGAGTTTGATCMTGGCTCAG |
| RE.A710 | CAAGCAGAAGACGGCATACGAGATTAGCAGACAGTCAGTCAGCCGAGTTTGATCMTGGCTCAG |
| RE.A711 | CAAGCAGAAGACGGCATACGAGATTCATAGACAGTCAGTCAGCCGAGTTTGATCMTGGCTCAG |
| RE.A712 | CAAGCAGAAGACGGCATACGAGATTCGCTATAAGTCAGTCAGCCGAGTTTGATCMTGGCTCAG |
| RE.B701 | CAAGCAGAAGACGGCATACGAGATAAGTCGAGAGTCAGTCAGCCGAGTTTGATCMTGGCTCAG |
| RE.B702 | CAAGCAGAAGACGGCATACGAGATATACTTCGAGTCAGTCAGCCGAGTTTGATCMTGGCTCAG |
| RE.B703 | CAAGCAGAAGACGGCATACGAGATAGCTGCTAAGTCAGTCAGCCGAGTTTGATCMTGGCTCAG |
| RE.B704 | CAAGCAGAAGACGGCATACGAGATCATAGAGAAGTCAGTCAGCCGAGTTTGATCMTGGCTCAG |
| RE.B705 | CAAGCAGAAGACGGCATACGAGATCGTAGATCAGTCAGTCAGCCGAGTTTGATCMTGGCTCAG |
| RE.B706 | CAAGCAGAAGACGGCATACGAGATCTCGTTACAGTCAGTCAGCCGAGTTTGATCMTGGCTCAG |
| RE.B707 | CAAGCAGAAGACGGCATACGAGATGCGCACGTAGTCAGTCAGCCGAGTTTGATCMTGGCTCAG |
| RE.B708 | CAAGCAGAAGACGGCATACGAGATGGTACTATAGTCAGTCAGCCGAGTTTGATCMTGGCTCAG |
