# Supplementary material for: Diversity, composition, and networking of saliva microbiota distinguish the severity of COVID-19 episodes as revealed by an analysis of 16S rRNA variable V1-V3 region sequences
Source: mSystems. 2023 Jun 13;8(4):e01062-22. doi: 10.1128/msystems.01062-22 (PMC10470033; doi:10.1128/msystems.01062-22)
Supplement: Table S2 — List of adapter primers. [file msystems.01062-22-s0007.docx]

| Name | Sequence 5´- 3´ |
| --- | --- |
| Seq_V3 | TATGGTAATTCAATTACCGCGGCTGCTGG |
| Seq_V1 | AGTCAGTCAGCCGAGTTTGATCMTGGCTCAG |
| ISP | CTGAGCCAKGATCAAACTCGGCTGACTGACT |
