# Supplementary material for: Diversity, composition, and networking of saliva microbiota distinguish the severity of COVID-19 episodes as revealed by an analysis of 16S rRNA variable V1-V3 region sequences
Source: mSystems. 2023 Jun 13;8(4):e01062-22. doi: 10.1128/msystems.01062-22 (PMC10470033; doi:10.1128/msystems.01062-22)
Supplement: Table S3 — Species linked to S. pneumoniae. [file msystems.01062-22-s0008.docx]

| Group | Species |
| --- | --- |
| AC | *Acidipropionibacterium acidifaciens* (+)  *Acinetobacter baumannii* (+)  *Catonell morbi* (-)  *Gemella haemolysans* (+)  *Mogibacterium diversum* (-)  *Selemonas artemidis*(+)  *Veillonella parvula* (+) |
| AN | *Bergeyella sp*. HMT322 (+)  *Gemella sanguinis* (+)  *Granulicatella adiacens* (+)  *Heamophilus parainfluenzae* (+)  *Leptotrichia sp. HMT225* (+)  *Streptococcus sanguinis* (+) |
| AP | *Gemella sanguinis* (+)  *Granulicatella adiacens* (+)  *Fretibacterium fastidiosum* (+) |
| HP | *Granulicatella adiacens* (+)  *Gemella sanguinis* (+)  *Letotricia wadei* (-) |
| DHP | *Streptococcus sanguinis* (+)  *Gemella sanguinis* (+)  *Leptotrichia sp* HMT417 (+)  *Abiotrohia defectiva* (+)  *Prevotella multisaccharivorax* (-)  *Mycoplasma orale* (-) |

(+) Positive link; (-) Negative link
