# Supplementary material for: Diversity, composition, and networking of saliva microbiota distinguish the severity of COVID-19 episodes as revealed by an analysis of 16S rRNA variable V1-V3 region sequences
Source: mSystems. 2023 Jun 13;8(4):e01062-22. doi: 10.1128/msystems.01062-22 (PMC10470033; doi:10.1128/msystems.01062-22)
Supplement: Legends — to supplemental figures and table. [file msystems.01062-22-s0009.docx]

**Text to supplementary files**

Supplementary Figure 1. Pairwise comparison between clinical groups analyzed with the enhanced Volcano test. AN vs AC) comparison between the ambulatory SARS-CoV-2 negative group and the asymptomatic control group; AP vs AN) comparison between the ambulatory SARS-CoV-2 positive and the ambulatory SARS-CoV-2 negative. Species in orange dots presented increased abundance and those in blue decreased abundance.

Supplementary Figure 2. Random forest ROC Curve. Groups are indicated to the right of the figure, AC asymptomatic, AN ambulatory SARS-CoV-2 negative, AP ambulatory SARS-CoV-2 positive, H hospitalized, HD deceased patients.

Supplementary Figure 3. Boxplots of the Log-transformed relative abundance of species belonging to the core-microbiome, with over 1.0% abundance in at least 50% of all saliva samples in patients from all clinical groups.

Supplementary Figure 4. A) Bean plot and ROC Curve. For each plot, the width of the distribution shows the frequency of samples at a given value of the dysbiosis score. The bean diagrams show that the average distance of the AC microbiota is less than the rest of the groups. B) The ROC plot and AUC values are shown.

Supplementary Figure 5. Stacked bar graphs describing the mean of bacterial composition per clinical group at the taxonomic level of family (A) and of species (B), in order to appreciate the difference in information on the microbial communities when results are described at different taxonomic level.

Supplementary Table 1. List of primers used for the amplification of the hypervariable regions V1-V3 of the 16S gene.

Supplementary Table 2. List of primers used as adapters in the sequencing of the V1-V3 amplicons from the 16S gene, in the MiSeq instrument.

Supplementary Table 3. Species showing links with *Streptococcus pneumoniae* in the clinical groups of patients with COVID-19 and in asymptomatic individuals.
